# The Role of Oral Antivirals for COVID-19 Treatment in Shaping the Pandemic Landscape

**DOI:** 10.3390/jpm12030439

**Published:** 2022-03-11

**Authors:** Cleo Anastassopoulou, Sophia Hatziantoniou, Fotini Boufidou, George P. Patrinos, Athanasios Tsakris

**Affiliations:** 1 Department of Microbiology, Medical School, National and Kapodistrian University of Athens, 75 Mikras Asias Street, 11527 Athens, Greece; atsakris@med.uoa.gr; 2 Laboratory of Pharmaceutical Technology, Department of Pharmacy, School of Health Sciences, University of Patras, 26504 Patras, Greece; sohatzi@upatras.gr; 3 Neurochemistry and Biological Markers Unit, 1st Department of Neurology, Medical School, National and Kapodistrian University of Athens, 11528 Athens, Greece; fboufidou@med.uoa.gr; 4 Laboratory of Pharmacogenomics and Individualized Therapy, Department of Pharmacy, School of Health Sciences, University of Patras, 26504 Patras, Greece; gpatrinos@upatras.gr; 5 Zayed Center for Health Sciences, United Arab Emirates University, Al-Ain P.O. Box 15551, United Arab Emirates; 6 Department of Genetics and Genomics, College of Medicine and Health Sciences, United Arab Emirates University, Al-Ain P.O. Box 15551, United Arab Emirates

Several vaccines against coronavirus disease 2019 (COVID-19) were developed and made available in a record time, just over a year after the outbreak of severe acute respiratory syndrome coronavirus 2 (SARS-CoV-2). These vaccines, especially those based on the novel mRNA technological platform, have contributed substantially to mitigating the devastating effects of the pandemic that has disrupted socio-economic activities around the globe and claimed the lives of more than 5.9 million people as of 3 March 2022 [1]. As with most prophylactic vaccines, however, COVID-19 vaccines do not provide sterilizing immunity, meaning that they do not totally prevent infection (and thus virus transmission, disease, and death). Hence, non-pharmaceutical interventions, such as wearing face masks, social distancing, and the ventilation of indoor spaces, along with mass vaccination, currently form the pillars of our armamentarium against SARS-CoV-2. Until recently, our arsenal lacked antivirals, aside from bespoke antibodies, which were authorized for use less than eleven months after the release of the SARS-CoV-2 sequence.

Monoclonal antibodies (mAbs), designed to block the virus’ spike protein-mediated attachment and entry into human cells—such as Regeneron’s REGEN-COV (Casirivimab with Imdevimab) and Lilly’s Bamlanivimab and Etesevimab cocktail—were shown to reduce viral replication and the hospitalization of patients [2,3]. mAbs have generally proven highly effective in the early stages of COVID-19, but ineffective against certain SARS-CoV-2 variants, in particular the Beta and Gamma variants, as well as Omicron (B.1.1.529), which is extensively mutated in its spike protein [4]. GSK’s Sotrovimab and AstraZeneca’s Evusheld (AZD7442) reportedly remained effective against this first Omicron variant (BA.1) [5]. Nevertheless, a second Omicron variant (BA.2) emerged and is currently spreading rapidly across the globe. BA.2 contains the BA.1 changes plus an additional six substitutions and three deletions, three of which lie in the receptor-binding domain (RBD). Importantly, this latest variant is not neutralized with detectable titers by any of the therapeutic mAbs, including Sotrovimab and Evusheld [6], raising concerns regarding the ability of SARS-CoV-2 to swiftly evade current antibodies, either monoclonal or polyclonal, and possibly reach pan-resistance to these therapies in the very near future [4]. 

Apart from therapeutic antibodies, which may have to be re-designed to target better-conserved viral elements in anticipation of the next evolutionary direction of SARS-CoV-2, so far only convalescent plasma (from recovered patients) and repurposed drugs have been used as antivirals, and with little success. Potential therapeutic strategies for COVID-19 were reviewed by Ferreira et al. early in the course of the pandemic [7]. As emphasized by Griffin et al., an appreciation of the different disease stages, which essentially guide the timing for the administration of various agents, is critical for advancing therapeutics for the treatment of COVID-19 [8]. Identified stages of COVID-19 include three periods (pre-exposure, incubation, and detectable viral replication) and five phases: the viral symptom phase, early inflammatory phase, secondary infection phase, multisystem inflammatory phase, and tail phase. This latter phase is more commonly known as “long COVID” [8]. Accordingly, antivirals are likely to provide benefits against COVID-19 if given early in the course of disease, as soon as possible after COVID-19 diagnosis and within five days of symptom onset. Early intervention depends on access to early and reliable testing.

This optimal timing seems to also hold for remdesivir, as shown by a recently published randomized controlled trial; a three-day course of remdesivir in newly diagnosed non-hospitalized patients who were at high risk for COVID-19 progression resulted in an 87% lower risk of hospitalization or death compared to placebo [9]. After securing an emergency use authorization (EUA) in May 2020, remdesivir (GS-5734, brand name “Veklury”, Gilead Sciences Inc., Foster City, CA, USA) was the first, and so far the only, antiviral repurposed for SARS-CoV-2 that has received full approval from the Food and Drug Administration (FDA). The FDA approved remdesivir in October 2020, but only for adults and select pediatric patients with severe COVID-19 who required hospitalization. The drug was found to shorten the recovery time of people hospitalized with COVID-19 and lower respiratory tract infection from 15 days to 11 [10]. As of 21 January 2022, remdesivir can be provided to patients with mild-to-severe COVID-19 who are not yet hospitalized if they are at high risk of severe disease.

The parent molecule of remdesivir, GS-441524, showed the ability to target emerging RNA viruses, such as SARS-CoV and MERS-CoV of the *Coronaviridae* or the Zika and dengue viruses of the *Flaviviridae* family [11]. The drug was also tested against ebolavirus (EBOV) in the first randomized controlled clinical trial (RCT), which was stopped after an interim analysis that showed an inferiority of remdesivir to treatments with mAbs (MAb114 and REGN-EB3) [12]. Remdesivir is a prodrug of a nucleoside analog with direct antiviral activity against several single-stranded RNA viruses [13]. In order to transform to their active metabolites that effectively inhibit viral RNA replication, nucleoside analogs require intracellular activation by phosphorylation. The phosphorylated synthetic compounds compete with endogenous natural nucleoside pools for incorporation into replicating viral RNA, and the incorporation of the analog molecule disrupts RNA elongation and subsequent molecular processes. 

Remdesivir-triphosphate (remdesivir-TP) competes with ATP for incorporation into new strands [13]. In SARS-CoV-2, remdesivir-TP causes the termination of RNA synthesis at three positions after the position where it is incorporated (*i* + 3), as revealed by a recent biochemical analysis [14]. The high selectivity of remdesivir over the incorporation of its natural nucleotide counterpart ATP was not shared by the triphosphate forms of 2′-C-methylated compounds, including sofosbuvir, which is approved for the management of hepatitis C virus (HCV) infection and the broad-acting antivirals, favipiravir and ribavirin [14]. Remdesivir is thus a nucleotide pro-drug inhibitor of the viral RNA-dependent RNA polymerase (RdRp), on which the replication of SARS-CoV-2 depends. Nevertheless, its widespread use is limited due to the need for intravenous infusion, which constitutes a pragmatic challenge for implementing outpatient treatment. Gilead and other manufacturers are pursuing oral formulations of remdesivir, which remains potent against the Omicron variant, as reported by Gilead [15].

Therapeutic agents that can be administered orally are much easier to implement in the outpatient setting. The recent EUA of two new oral antivirals, Merck’s Molnupiravir and Pfizer’s Paxlovid, is expected to have a profound impact on the pandemic, predominantly by keeping vulnerable patients out of the hospital, thereby relieving the pressure from overwhelmed healthcare professionals and systems worldwide, especially in countries with fragile national health care systems and limited resources [16]. However, why were therapeutics not given priority in strategic planning? Was it not possible to have developed antivirals sooner, in parallel with COVID-19 vaccines? One cannot help but wonder whether their earlier availability could have largely prevented many of the catastrophic consequences of the pandemic. However, how do these new oral antiviral drugs work and are they entirely novel?

Molnupiravir originated from George Painter’s laboratory and Drug Innovations at Emory University [17]. In 2013, efforts were made to develop a broad-acting antiviral for RNA-encoded viruses, including highly pathogenic coronaviruses and influenza viruses, as well as encephalitic alphaviruses such as Venezuelan, Eastern, and Western equine encephalitis viruses. To this end, nucleoside analogs were explored because of their potency, high genetic barrier to resistance, and oral availability. An *in vitro* activity of molnupiravir was also found against SARS-CoV-2 in human airway epithelial cell cultures in the early stages of the pandemic [18]. The therapeutic was then licensed to Ridgeback Biotherapeutics, and just two months later, Merck secured exclusive worldwide rights to develop and commercialize the drug (under the brand name “Lagevrio”). 

Molnupiravir (also known as EIDD-2801/MK-4482) is a prodrug of the active antiviral ribonucleoside analog β-d-*N*^4^-hydroxycytidine (NHC; EIDD-1931), which after distribution into various tissues, is converted by host kinases into EIDD-1931 5′-triphosphate, the active antiviral agent [19]. Molnupiravir exerts its antiviral action through the introduction of copying errors during viral RNA replication, and not by terminating the RNA chain elongation, as is the case for remdesivir. More specifically, NHC, the active form of molnupiravir, is used as a substrate instead of cytidine triphosphate (CTP) or uridine triphosphate (UTP) by the viral RdRp. When the RdRp uses the resulting RNA as a template, NHC directs the incorporation of either G or A, leading to mutated RNA products [20]. NHC can form stable base pairs with either G or A in the RdRp active center, explaining how the polymerase escapes proofreading and synthesizes mutated RNA. Over cycles, mutations accumulate, leading to viral ‘error catastrophe’. This two-step mutagenesis mechanism seems to apply to polymerases of other viruses, thereby explaining the broad-spectrum antiviral activity of molnupiravir [20].

Lethal mutagenesis leads to the inhibition of viral infectivity. Indeed, NHC was shown to block SARS-CoV-2 transmission in ferrets [21]. Merck is currently testing whether its drug can be used prophylactically. Molnupiravir also inhibits SARS-CoV-2 replication in human lung tissue and reduces SARS-CoV-2 RNA in patients [22]. On 30 November 2021, an FDA advisory committee voted narrowly—13 to 10—in favor of an EUA for molnupiravir. Phase III results from 1433 patients showed that the drug reduced hospitalization or death by 30% when it was administered within five days of symptom onset, while interim results of Phase II/III data from the ongoing MOVe-OUT trial showed a 48% reduction [17,23]. The study included patients recruited from multiple countries with risk factors of poor disease outcome. In the first part of the trial, the drug reduced hospitalization or death by about 50%, compared with the placebo in patients with mild-to-moderate COVID-19, when it was administered within five days of symptom onset: 7.3% of patients who received molnupiravir were either hospitalized or died through day 29 (28/385), compared with 14.1% of placebo-treated patients (53/377). Eight deaths were recorded in the placebo arm through day 29, while no deaths were reported in the molnupiravir arm during the same period.

Key concerns regarding the use of molnupiravir include the real risk for the mutagenicity of host cells and the theoretical potential for the acceleration of the virus’s evolution, particularly in immunocompromised patients [17,24]. In addition, molnupiravir may affect bone and cartilage growth; therefore, it is not authorized for use in patients younger than 18 years of age. Paxlovid’s profile and efficiency appear to be much more favorable. 

Paxlovid is comprised of nirmatrelvir, a SARS-CoV-2 main protease inhibitor (M^pro^: also referred to as 3CL^pro^ or nsp5 protease) with potent pan-human-coronavirus activity *in vitro*, co-packaged with ritonavir, an HIV-1 protease inhibitor and CYP3A inhibitor. Although ritonavir has no activity against SARS-CoV-2 on its own, it is included in low doses (100 mg), in order to inhibit the CYP3A-mediated metabolism of nirmatrelvir and, consequently, increase nirmatrelvir plasma concentrations to levels that are anticipated to inhibit SARS-CoV-2 replication; thus, ritonavir is used as a pharmacokinetic enhancer in paxlovid [25,26]. Nirmatrelvir (PF-07321332) works by covalently binding to cysteine residues in the active site of the 3-chymotrypsin-like protease or main protease of SARS-CoV-2, inactivating this essential viral enzyme (with no recognized human analogs) that is required to process precursor proteins into functional products [25,27]. Such protease inhibitors were successfully developed and are approved for the treatment of HIV/AIDS and HCV, while other SARS-CoV-2 M^pro^-targeting compounds are in development. Targeting conserved viral elements that are less tolerant to change is likely to maintain antiviral activity, in contrast to vaccines and mAbs that target the spike protein.

Nirmatrelvir inhibits M^pro^ activity and viral replication in cell cultures and in a mouse model when administered orally [25]. Added substructures from the HCV protease inhibitor boceprevir and additional optimizations ensure the oral bioavailability of this drug component, which is also available in an intravenous form [17]. In a phase I trial in four healthy participants, the drug was safe and well-tolerated, reaching levels greater than needed to inhibit viral replication in cell cultures [28]. The interim analysis of a phase II/III randomized, placebo-controlled study of paxlovid in symptomatic, unvaccinated, non-hospitalized adult patients with COVID-19, who were at risk for severe illness (the EPIC-HR trial, Evaluation of Protease Inhibition for COVID-19 in High-Risk Patients), showed that the drug reduced the risk of hospitalization or death by 89.1% [28,29]. Of the patients who received the drug within three days of symptom onset, 0.8% (3/389) were admitted to hospital up to day 28 with no deaths, compared with 7% (27/385) hospitalized and 7 deaths in the placebo group. Similar results were obtained in the group of patients treated within five days of symptom onset, with 1% (6/607) in the paxlovid arm admitted up to day 28 (no deaths) and 6.7% (41/612) in the placebo group (10 deaths, 1.6%). Efficacy was maintained at 88.9% and 87.8% among patients commencing treatment within three days and within five days after symptom onset, respectively, with zero deaths occurring in the group that received nirmatrelvir plus ritonavir and 13 deaths occurring in the placebo group, without evident safety concerns in the final analysis [28].

A limitation in the use of paxlovid is that its concomitant administration with certain commonly prescribed drugs, such as statins or anticoagulants, may result in potentially important drug interactions. Such interactions would need to be managed, by first weighing risks and benefits, through the temporary discontinuation of concomitant medications, avoidance of co-administration, dose reduction/use of alternative concomitant medication, or increased monitoring of adverse events or concomitant medication drug levels.

Omicron heralded a turning point in the COVID-19 pandemic. Humanity should be prepared for additional epidemic waves of new and better-adapted SARS-CoV-2 variants that will not necessarily be more benign than their predecessors. The prevention of severe COVID-19 disease through vaccines, preferably with expanded targets beyond the spike protein, remains a priority; however, antivirals, particularly agents, which can be administered orally to keep vulnerable patients and those refusing to be vaccinated out of hospital, are an important tool in our armamentarium. Hopefully, these two new oral antivirals—and those that are likely to follow—will help break the Sisyphean circle of vaccine boosters, decreasing humoral immunity, infections and reinfections, and shifting the pandemic landscape to a new normalcy of coexistence with SARS-CoV-2.

## Data Availability

Not applicable.
